# The effectiveness of remote computerized cognitive training for older adults with mild cognitive impairment: A systematic review

**DOI:** 10.1177/20552076261421682

**Published:** 2026-02-11

**Authors:** Ana Isabel Martins, Anabela G. Silva, Joana Pais, Nelson P. Rocha

**Affiliations:** 1 Institute of Electronics and Informatics Engineering of Aveiro (IEETA), Department of Medical Sciences, 56062 University of Aveiro, Aveiro, Portugal; 2 RISE-Health, School of Health Sciences, 56062 University of Aveiro, Aveiro, Portugal; 3 EPIUnit – Institute of Public Health, Laboratory for Integrative and Translational Research in Population Health (ITR), 26706 University of Porto, Porto, Portugal

**Keywords:** Remote computerized cognitive training, mild cognitive impairment, cognitive function, RCCT, older adults

## Abstract

**Background:**

Remote computerized cognitive training (RCCT) is increasingly used as a scalable intervention to support cognitive function in older adults, particularly those with mild cognitive impairment (MCI). However, evidence regarding its effectiveness remains unclear.

**Objective:**

This study aimed to identify and critically assess the evidence on the effectiveness of RCCT in improving the cognitive function of older adults with MCI.

**Methods:**

A systematic search was conducted in PubMed, Scopus, and Web of Science to identify randomized controlled trials evaluating RCCT in older adults with MCI. Screening of references was performed against predefined inclusion criteria. Data extraction and the methodological quality of included studies using the Physiotherapy Evidence Database scale were performed by two authors.

**Results:**

A total of 17 studies were included. Among the eight studies comparing RCCT to leisure-based activities, statistically significant differences favouring RCCT were reported in 11 out of 25 cognitive assessments. In comparison with usual care (5 studies), 9 out of 16 assessments showed significant differences. When RCCT was compared to no intervention (4 studies), statistically significant differences were found in 6 out of 8 assessments. Overall, the findings were inconsistent, and the evidence remains inconclusive. High variability was found in intervention content, duration, control conditions, and outcome measures.

**Conclusions:**

The results were unclear regarding the effectiveness of RCCT. Clearer definitions of the intervention and higher methodological quality are needed.

**Trial registration:**

PROSPEROCRD42023444763

## Introduction

Mild cognitive impairment (MCI) is a common condition among older adults, characterized by a decline in cognitive function, and associated with a higher risk of developing dementia.^1^ Although patients with MCI typically have some intellectual ability and daily life skills preserved.^2^ This is a progressive condition considered an intermediate stage between normal aging and dementia.^3^ Recent evidence from a systematic review and meta-analysis indicates that the global pooled prevalence of MCI among older adults is approximately 19.7%.^4^ Given the growing aging of the population worldwide, finding accessible and effective strategies to target cognitive decline has become a pressing priority.^5^

Cognitive training, which consists of engaging in structured activities designed to challenge specific cognitive abilities, has shown promise in preserving cognitive function in older adults.^6–8^ Cognitive training is particularly important for individuals with MCI, as it enhances cognitive function through repeated practice of task-specific exercises, stimulating different cognitive domains based on individual needs, such as memory, attention, and executive functioning.^9^ Cognitive training can potentially slow the progression of cognitive decline, allowing individuals to maintain independence and quality of life.^9^

Cognitive training is usually performed in the presence of a health professional who can take advantage of digital means.^7,10–13^ However, in recent years, remote computerized cognitive training (RCCT) has emerged as a flexible and scalable alternative to traditional in-person cognitive training. It refers to cognitive training delivered through digital platforms that allow users to engage in training sessions remotely without the need for synchronous supervision by a health professional.^14^

RCCT offers the flexibility of performing cognitive training at any time from the comfort of the individual's homes.^7,10^ In addition, RCCT is usually administered using interactive and visually attractive interfaces and algorithms that allow for the immediate adjustment of the training content and difficulty level based on each user's performance.^6,15^ RCCT makes it easier to reach populations with limited access to in-person interventions, such as older adults who live far from urban centres or have disabilities that compromise travelling to a health centre.^16^ The use of technologies for cognitive training also allows for the incorporation of elements of fun and excitement, which can help minimize poor adherence, often seen in traditional training methods.^6^

In this study, we aim to focus exclusively on digital computerized solutions for RCCT with asynchronous supervision. To the best of our knowledge, no synthesis of evidence is available on the effectiveness of RCCT in older adults with MCI. To address this gap, the present systematic review aims to identify and critically assess the evidence on the effectiveness of RCCT in older adults with MCI. By synthesizing findings from existing studies, this review seeks to provide a clearer understanding of the potential benefits, limitations, and future directions for RCCT interventions in older adults with MCI.

## Methods

### Registration of systematic review

The preferred reporting items for systematic reviews and meta-analyses (PRISMA) guidelines were followed in this review,^17^ and the protocol was registered at the International Prospective Register of Systematic Reviews (PROSPERO; CRD42023444763).

### Search strategy and eligibility

The following databases were searched: PubMed, Scopus, and Web of Science using the following Boolean expression: TITLE-ABSTRACT-KEY ((computer OR ‘Virtual Reality’ OR ‘Serious Games’ OR web-based OR mobile) AND (‘Cognitive training’ OR ‘Cognitive rehabilitation’) AND (‘randomized controlled trial’ OR RCT)).

Pubmed was searched using MeSH terms and filters for age and article type (to exclude reviews, systematic reviews, and books). No date limit was used, but languages of publication were limited to English. The full search strategy for PubMed is provided in Supplementary Table 3. The list of references of all included studies was checked for additional references not identified by electronic searches. To be included in this systematic review, studies had to: (i) include a sample of older adults (mean age ≥65 years old) with a diagnosis of MCI associated with any underlying pathology or to no specific pathology; (ii) focus on RCCT, i.e. cognitive training delivered through digital platforms that allow users to engage in training sessions remotely without the need for synchronous supervision by a health professional; (iii) compare the RCCT against cognitive training performed face-to-face, with or without technology, leisure-based activities, usual care, or no intervention; (iv) report on cognitive function outcomes assessed by any validated instrument (e.g. Mini-Mental State Examination (MMSE), Montreal Cognitive Assessment (MoCE)), and v) be a randomized controlled trial or quasi-randomized trial.

The database search was conducted in January 2025 and aimed to include all references published since each database's inception until 31 December 2024.

### Study selection

The analysis and selection of the studies were performed in three steps. First, duplicated references, references with no abstract, and those not written in English were removed. Then, titles and abstracts were screened for inclusion, and those not meeting the inclusion criteria were removed. Finally, the full text of the remaining references was retrieved and checked against the inclusion criteria. The reference list of the included articles was checked, and potentially eligible studies that may have been missed during the initial search were identified. Throughout this process, all references were screened by two authors, and any disagreements were discussed and resolved by consensus. Authors were contacted whenever an article was unavailable and potentially relevant.

### Data extraction

Concerning data extraction, the following information was registered in a customized data sheet for each of the studies included in the review: author, date, study design, sample size, participant demographics (age, sex), and type of technology used (e.g. web-based application, virtual reality, etc.). Comparison (e.g. RCCT using technology vs. face-to-face cognitive training), intervention description and duration, outcomes, and results. The authors were contacted when the information to be extracted was not available.

### Methodological quality assessment

The methodological quality of included studies was assessed using the Physiotherapy Evidence Database (PEDro) scale,^18^ which is considered a reliable scale.^18^ Each manuscript was independently evaluated by a pair of two authors from a group of three (AIM, AGS, and NPR). Disagreements were resolved through discussion between each pair of authors until a consensus or, if necessary, by consulting the third author. The PEDro Scale consists of 11 items, each receiving a score of 1 if present or 0 if absent. The first item is excluded from the overall score, resulting in a maximum possible score of 10. The total score reflects methodological quality, categorized as ‘poor’ for scores of 0 to 3, fair for scores of 4 to 5, good for scores of 6 to 8 and excellent for scores of 9 and 10.^19^ The percentage of agreement between the assessors was calculated for each item of the scale.

### Data synthesis and analysis

A PRISMA flowchart was used to show the progression of the studies through the review.

The results of the systematic review were presented in a narrative form. The data analysis was conducted by cognitive function, including global cognition, attention, executive functions, language, visuo-constructional function, processing speed, and memory. The same study could be included in the analysis of more than one domain (if measuring more than one cognitive domain). The classification per cognitive domain was based on what each article's authors reported. However, in some cases, the authors of the included papers indicated using the same instrument to evaluate different cognitive domains. In these situations, the domain selection was made by consulting a panel of neuropsychology experts with an academic degree in neuropsychology and more than 5 years of field experience (n = 4). The description of the studies was organized first by cognitive domain and then by comparator. A meta-analysis was not possible because we had few studies within each cognitive domain, and the comparators were highly diverse.

## Results

### Study selection

A total of 1739 references were identified. In the first step, 722 duplicates and 20 references with no abstract were excluded. After that, 997 references remained for screening based on the title and abstract. Of these, 918 were excluded because they did not meet the eligibility criteria, whereas 78 full-text articles from the initial database and two resulting from the included articles’ references were retrieved for full-text screening. After that, another 62 articles were excluded due to not having a sample of older adults (mean ≥65 years old), not assessing cognitive function, not including participants with MCI, or not reporting on RCCT. Seventeen articles corresponding to 17 studies were included in this systematic review. Five authors were contacted to request full-text articles and three were contacted for missing information; however, no responses were received.

Altogether, the included studies comprised 1236 participants, with a pooled mean age of 72.87 years (SD = 4.7).

**Figure 1.**
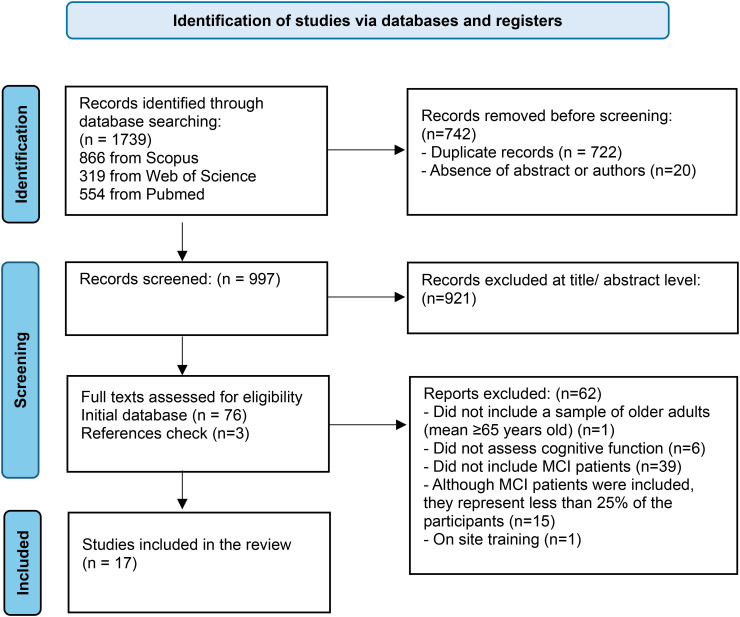
PRISMA flow diagram. PRISMA: preferred reporting items for systematic reviews and meta-analyses.

The flowchart of this review is presented in Figure 1.

### General overview of included studies

The studies reviewed include a range of RCCT technologies. Ten studies used web-based programs: BrainHQ was employed in seven studies, Lumosity was used in two, while Cogmed, and one unspecified program were each used in one study. Tablet applications were used in three studies (USMART, Neuro-World, and the Mobile Intelligent Cognitive Training Application). Desktop software (RehaCom) and a Virtual Reality Rehabilitation System (VRRS, Khymeia) were each used in one study. Another study did not report the type of technology used. Table 1 describes the RCCT technologies used in each study.

**Table 1.** Description of the RCCT technologies.

Study authors (year)	Name of the technology	Description of the RCCT	Technology type
Barnes et al. 2008^20^	BrainHQ	Involved seven exercises that were designed to improve processing speed and accuracy in the auditory cortex; primary and working auditory memory tasks were woven implicitly into the exercises.	Web-based application
Rosen et al. 2011^21^	BrainHQ	Involved seven exercises designed to improve processing speed and accuracy in auditory processing.	Web-based application
Finn et al. 2017^22^	Lumosity	Had four broad cognitive domains targeted: nominally attention, processing speed, visual memory, and cognitive control. Training sessions contained four or five cognitive exercises.	Web-based application
Hyer et al. 2016^23^	Cogmed	Focused on working memory training, specifically on the ability to hold and manipulate information for short periods.	Web-based application
Lin et al. 2016^24^	BrainHQ	Included a Vision-based speed of processing (VSOP) training which includes five training tasks: Eye for detail, Peripheral challenge, Visual sweeps, Double decision, and Target tracker.	Web-based application
Wu et al. 2017^25^	BrainHQ	Consisted of five visual attention and information processing exercises. Exercises follow a defined order optimizing fidelity to the intervention.	Website-based application
Han et al. 2017^26^	USMART	Included a memory training program consisting of recalling a given set of words within 12 min in two consecutive sessions, the number of words to be memorized within a session was automatically sequentially increased in the following session from one to five words.	Tablet application
Nousia et al. 2021^27^	RehaCom	Consisted of a specially designed input panel and a large screen that helps patients be trained in several cognitive domains, such as episodic and delayed memory, verbal memory, attention, processing speed and executive function.	Desktop application
Li et al. 2019^28^	Not reported	Comprised of the following eight tasks: visual working memory task; 30-s memory task; episodic memory task; speed of calculation task; visual search task; alertness task; mental rotation task and an image re-arrangement task.	Web-based application
Manenti et al. 2020^29^	Virtual reality tele-rehabilitation system Khymeia	Included 12 exercises designed to enhance memory, visuospatial abilities, attention, and executive functions. In each treatment session, a participant worked with six exercises, 10 min each, task difficulty adaptively progressed, and the performances were continuously monitored by the therapist. The subject was asked to continue to perform each task until the end of the set time.	Virtual reality application
Duff et al. 2021^30^	BrainHQ	Consisted of an evolving suite of more than 25 web-based training tasks that seek to improve attention, processing speed, memory, intelligence, spatial abilities, and interpersonal skills.	Web-based application
Lin et al. 2021^31^	BrainHQ	Included five training paradigms (Eye for detail, peripheral challenge, visual sweep, double decision, and target tracker) that practice processing speed and attention. All exercises share visual components and focus on accuracy and fast reaction times.	Web-based application
Lin et al. 2020^32^	BrainHQ	Consisted of five exercises that target processing speed and attention. All tasks shared visual components, and the tasks became increasingly more difficult as subjects’ training progressed, thus requiring faster reaction times.	Web-based application
Baik et al. 2023^33^	Neuro-World	Trains attention, visual perception, memory, and executive functions.	Tablet application
Brill et al. 2024^34^	Not reported	Consisted of 16 different training games, each specifically training one or multiple cognitive domains (episodic memory, semantic memory, visuospatial abilities, working memory), thus facilitating the near transfer effect.	Not reported
Han et al. 2024^35^	Mobile Intelligent Cognitive Training Application	Included six rehabilitation modules: memory, attention, execution, calculation, thinking, and perception, with different difficulty levels set for cognitive training tasks in each module. During the training process, the application automatically adjusted the task difficulty level from easy to difficult according to the patient's training.	Tablet application
Devanand et al. 2022^36^	Lumosity	Included 6 modules randomly selected from 18 available modules that included memory tasks, matching tasks, spatial recognition tasks, and processing speed tasks.	Web-based application

RCCT: remote computerized cognitive training.

The interventions varied greatly in frequency and duration. The frequency of sessions varied from once a week to five times a week, and the sessions ranged from 30 to 160 min per session, and the total duration of the interventions ranged from 4 weeks to 18 months.

The control group included no intervention (n = 2), usual care (n = 5), and leisure-based activities (n = 8), which consisted of leisure-based cognitive activities such as crossword puzzles and sudoku. Also, two studies fail to report the control group intervention or the absence of intervention and another study reported that the control group received no intervention for 4 of 10 weeks, and RCCT in the remaining 6 weeks(n = 1). Table 2 describes the interventions for the RCCT and control group.

**Table 2.** Sample characterization and description of the interventions for the RCCT and control group.

Study	Characteristics of participants			Intervention		
Authors (year)	Sample size	Mean age (SD)	Female n (%)	RCCT group	Control group	Duration
Barnes et al. 2008^20^	EG (22) CG (25)	EG: 74.10 (8.7) CG: 74.80 (7.2)	EG: 9 (40.9) CG: 10 (40)	RCCT for 100 min per day, 5 days per week for 6 weeks.	Passive computer activities (reading, listening, visuospatial game) for similar amounts of time.	6 weeks
Rosen et al. 2011^21^	EG (6) CG (6)	EG: 70.67(10.58) CG: 78.00(7.92)	Not reported	RCCT for 100 min per day, 5 days per week until either achievement of asymptotic performance levels over a several-day period or completion of 80% of the training material in a given exercise. Training lasted an average of 2 months across participants.	Weekly ‘assignments’ that involved listening to audiobooks, reading online newspapers, and playing a visuospatially oriented computer game (Myst) for 30 min each, for a total of 90 min per day, 5 days per week. Training lasted an average of 2 months across participants.	8 weeks
Finn et al. 2017^22^	EG (12) CG (13)	EG: 69.00(7.69) CG: 76.38(6.47)	EG: 5 (62.5) CG: 3 (37.5)	4 to 5 sessions of training per week in a total of 30 training sessions, each containing four or five cognitive exercises.	Waitlist group.	4 to 8weeks
Hyer et al. 2016^23^	EG (34) CG (34)	EG: 75.10(7.4) CG: 75.20(7.8)	EG: 17 (50) CG: 19 (55.9%)	25 sessions completed over a 5- to 7-week period for approximately 40 min per day. The exercises were divided into eight different tasks each day that selected from a bank of 13 tasks.	The comparison condition involved the same training program as Cogmed without adaptability. The difficulty level remained constant across the entire intervention.	5 to 7weeks
Lin et al. 2016^24^	EG (10) CG (11)	EG: 72.9(8.2) CG: 73.1(9.6)	EG: 5 (50) CG: 6 (54.5)	Participants trained 1 h per day, 4 days per week, for 6 weeks in their homes by identifying what object they saw or where they saw it on the screen. The training automatically adjusted the task difficulty and speed based on the participant's performance.	1 h per day, 4 days per week for 6 weeks of online crossword, Sudoku, and solitaire in their homes.	6 weeks
Wu et al. 2017^25^	EG (40) CG (20)	EG: 66.5(8.9) CG:66.7(7.81)	Not reported	RCCT for 1 h a day, 5 days a week for 8 weeks.	Usual care.	8 weeks
Han et al. 2017^26^	EG (43) CG (42) Crossover	All participants: 74.01 (5.53)	20 (46.5)	RCCT for 30 min per session, twice a week, over the 4 weeks.	Usual care.	4 weeks
Nousia et al. 2021^27^	EG (25) CG (21)	EG: 71.20(5.07) CG:71.90(6.24)	EG: 19 (76) CG: 16 (76.19)	RCCT for 15 weeks, administered twice a week for approximately 1 hour per session.	Usual care.	15 weeks
Li et al. 2019^28^	EG (80) CG (80)	EG: 69.5(7.3) CG:71.5(6.8)	Not reported for the whole sample	RCCT for 3–4 times (about 120–160 min training in total) per week for 6 months.	Not reported.	26 weeks
Manenti et al. 2020^29^	EG (18) CG (17)	EG: 75.3(3.3) CG: 78.1(4.1)	EG: 5 (27.78) CG: 10 (58.82)	Face-to-face cognitive VRRS (12 sessions of 60 min of individualized cognitive rehabilitation over 4 weeks) followed by tele-rehabilitation (36 sessions of home-based cognitive VRRS training, 3 sessions per week over 12 weeks).	Face-to-face cognitive treatment as usual (12 sessions of 60 min face-to-face cognitive treatment as usual).	12 weeks
Duff et al. 2021^30^	EG (55) CG (58)	EG: 74.9(6.3) CG: 74.9(5.8)	EG: 26 (47) CG: 25 (43)	RCCT for 40 h, in 45-min sessions, 4 to 5 days per week for about 12-13 weeks.	The active control training program consisted of 6, primarily visual, computerized games.	12/13 weeks
Lin et al. 2021^31^	EG (24) CG (25)	EG:86.75(5.26) CG: 86.04(6.10)	EG: 15 (62.5) CG: 16 (64)	A ‘multi-functional interactive computer system’ (MICS) for 5 h per week for 4 weeks + Computerized cognitive intervention (CCI) for 4 1 hour sessions per week for 6 weeks.	An inert control condition, consisting of nothing outside of the ordinary, was implemented for the first 4 weeks, and Computerized cognitive intervention (CCI) for 6 more weeks.	10 weeks
Lin et al. 2020^32^	EG (56) CG (28)	EG: 75,23(7,49)CG: 73,68(6,92)	EG: 24 (42.86) CG: 15 (53.6)	Four 1-h sessions per week 6-week of a processing speed and attention targeted intervention.	Four 1-h sessions per week 6-week of online crossword, Sudoku, and solitaire games.	6 weeks
Baik et al. 2023^33^	EG (25) CG (25)	EG: 67.08(7.93)CG: 65.64(8.54)	EG: 17 (68) CG: 17 (68)	Three times a week over an 8-week period for a total of 24 occurrences.	No intervention.	8 weeks
Brill et al. 2024^34^	EG (52) Active CG (52) Waitlist CG: (56)	EG: 71.9(5.99)Active CG:71.9 (7.05)Waitlist CG: 71.6 (5.83)	EG: 28 (68) Active CG: 25 (68) Waitlist CG: 31(55)	Three-month intervention comprising a total of 60 at-home sessions of RCCT, five per week and weekly on-site group meetings.	The active control group did a 3-month protocol of time-matched unspecific cognitive activation (watching documentaries) while matching the amount of social interaction to the intervention group with weekly group sessions after the baseline. The waitlist control group had no intervention.	12 weeks
Han et al. 2024^35^	EG (57) CG (55)	EG: 65.79(8.63) CG: 66.00(9.45)	EG: 15 (26.3) CG: 16 (29.1)	Post-Stroke Cognitive Impairment management intervention (including health education, routine care, and health management) for 30 min per day, 5 days per week, for 12 weeks + RCCT for 30 min per day, 5 days per week, over 12 weeks.	Patients received comprehensive management of Post-Stroke Cognitive Impairment, including health education, routine care, and health management. The management plan was set at 30 min per day, 5 days per week, for 12 weeks.	12 weeks
Devanand et al. 2022^36^	EG (51) CG (56)	EG: 71.1(8.50) CG: 71.3(9.1)	EG: 34 (66.7) CG: 28 (50)	Initial intensive, home-based computerized training for games consisted of four 30-min training sessions per week for 12 weeks. Subsequent booster training was composed of four 30-min sessions, completed over 1 week and occurring at weeks 20, 32, 42, 52, 64, and 78. During weeks 32, 52, and 78, participants completed three sessions at home and the fourth in clinic. During weeks 20, 42, and 64, participants completed all four sessions at home.	Home-based computerized Crosswords consisted of four 30-min training sessions per week for 12 weeks. Subsequent booster training was composed of four 30-min sessions, completed over 1 week and occurring at weeks 20, 32, 42, 52, 64, and 78. During weeks 32, 52, and 78, participants completed three sessions at home and the fourth in clinic. During weeks 20, 42, and 64, participants completed all four sessions at home.	78 weeks

RCCT: remote computerized cognitive training; VRRS: Virtual Reality Rehabilitation System.

### Methodological quality

Studies scored between 4 and 10, with 2 studies (11.8%) scoring between 4 to 5, suggesting fair quality, 12 studies (70.6%) scoring between 6 and 8, suggesting good methodological quality and 3 studies (17.6%) between 9 and 10, suggesting excellent quality. The item with the lowest mean score across studies was item 6, ‘Blinding of who administered the intervention’, with only two studies meeting this criterion, followed by item 5, ‘Blinding of subjects’, which was fulfilled by six studies. All studies met items 1 – ‘Eligibility criteria’, item 2 – ‘Random allocation’, and item 10 – ‘Statistics for between-group comparison’. The percentage of agreement among raters varied between 90% and 100% with an average of 93.8%. Table 2 of the appendix presents the methodological quality of the included studies.

### Effectiveness of remote computerized cognitive training

#### Global cognition

Nine studies assessed global cognition,^20,21,26,28,30,33–36^ of which three used the Repeatable Battery for the Assessment of Neuropsychological Status,^20,21,30^ three used the MMSE,^26,28,35^ three used the MoCA,^33–35^ one used both MMSE and MOCA,^35^ and one used the 11-item Alzheimer's Disease Assessment Scale Cognitive (ADAS-Cog) and the Neuropsychological composite.^36^

Of the nine studies that assessed global cognition, three (one rated fair quality and the others of good quality) reported statistically significant differences favouring the RCCT group. The first study involved a long-term intervention consisting of 3–4 weekly sessions of 120–160 min each over 6 months, though it did not report the activities undertaken by the control group.^28^ The second study consisted of a weekly intervention performed three times per week for 8 weeks, compared to a no-intervention control group.^33^ The third study consisted of a 5-week intervention of 30 min over 12 weeks, compared with usual care.^35^ The remaining five studies, which demonstrated good to excellent methodological quality, found no significant differences between groups when comparing the RCCT to a leisure-based activities^20,21,30,34^ or usual care.^26^ Another study with good methodological quality, found significant differences between groups, favouring the leisure-based activities group on the ADAS-COG, but no difference on the neuropsychological composite.^36^

Table 1 in the Appendix presents the assessment instruments used and provides the baseline, post-intervention, and follow-up values for global cognition and other cognitive domains (when applicable).

#### Attention

Attention was assessed in eight studies^20,21,22,25,27–29^^,33^ using a total of nine different measurement instruments, of which four studies found statistically significant differences favouring the RCCT.^22,27,28,33^ Of those, three studies with good methodological quality, compared the RCCT against no intervention^33^ usual care,^27^ and a waiting list,^22^ and the other, with fair quality, did not report the comparator.^28^

The remaining four studies, two with good to excellent methodological quality, compared RCCT to leisure-based activities,^20,21^ one with fair quality compared RCCT with usual care,^25^ and another with good quality compared RCCT with a face-to-face treatment,^29^ and all reported no statistically significant between-group differences.

#### Executive functions

Six studies assessed executive functions^20,22,23,28,31,33^ using seven different instruments. Only one^33^ of the six studies found statistically significant differences between-groups favouring the RCCT.

The study that reported statistically significant differences had good methodological quality and compared RCCT to no intervention.^33^

Of the five studies that reported no between-group differences, two (one with excellent methodological quality and the other with good quality) compared RCCT to leisure-based activities,^20,23^ one (with good methodological quality), compared RCCT with no intervention,^22^ one (with good methodological quality) compared the intervention (‘multi-functional interactive computer system’ for 4 weeks + computerized cognitive intervention for 6 weeks to 4 weeks of no intervention and 6 weeks of computer-based cognitive intervention^31^ and another one (with fair methodological quality) did not report the control condition.^28^

#### Language

Eight studies investigated the impact of RCCT on the language^20,21,24,27–29^^,33,34^ using a total of 12 different instruments, and, of those, three studies found statistically significant differences between-groups favouring the RCCT.

The three studies that reported statistically significant differences between groups in favour of the RCCT group all demonstrated good methodological quality. Two of these studies compared RCCT to usual care^29,27^ while the third compared it to no intervention.^33^

Of the five studies that did not find differences, four studies, with good to excellent methodological quality, compared a RCCT solution with leisure-based activities^20,24,21,34^ and another study with fair quality did not report the control condition.^28^

#### Visuo-Constructional function

Five different instruments were used to assess visuo-constructional function in six studies^20,21,27–29^^,34^ and, of those, three studies found statistically significant differences between-groups favouring the intervention group.

Of the three studies that found statistically significant differences between-groups favouring the RCCT group, two (with good methodological quality) compared the intervention to usual care^27,29^ and one (with fair methodological quality) did not report the control group intervention and found differences only on the Rey-Osterrieth Complex Figure Test but not in the visuospatial ability section of the Chinese version of Addenbrooke's Cognitive Examination-Revised.^28^

The three studies that reported no between-group differences presented excellent methodological quality and compared the RCCT to leisure-based activities.^20,21,34^

#### Processing speed

Five studies investigated the impact of RCCT on the processing speed cognitive domain^24,25,27,28,32^ using four different instruments (Useful Field of View, CNS Vital signs subtest, Trail Making Test B, and Symbol digit substitution test), and, of those, three studies found statistically significant differences between-groups favouring the intervention group.

Of the three studies that found statistically significant differences, two (with good methodological quality) compared the RCCT to leisure-based activities^24,32^ and the other (also with good methodological quality) compared it against usual care.^27^

The two studies that did not find differences between groups had both fair methodological quality and compared the RCCT to usual care^25^ and a non-reported control group condition.^28^

#### Memory

To assess memory, 29 instruments were used across 15 studies.^20,21,22–31^^,33–35^ Of the 15 studies, eight reported a significant difference favouring the RCCT,^20,21,23,26–28^^,33,35^ two reported a significant difference favouring the control group,^30,25^ and the remaining 5 did not find statistically significant differences between groups.

Of the eight studies that reported significant differences favouring RCCT, three compared it against leisure-based activities,^20,21,23^ three compared it against usual care, one against no intervention, and one did not report the control condition. Of the three studies that compared RCCT to leisure-based activities and reported statistically significant differences in favour of the RCCT group, one was rated as having good methodological quality^23^ and the other two were rated as having excellent methodological quality.^21,20^ The three studies that compared RCCT to a usual care control and reported statistically significant differences in favour of the RCCT group, demonstrated good methodological quality.^26,35,27^ The remaining two studies demonstrated good methodological quality, and one compared RCCT to a no-intervention control group^33^ while the other did not report the control group condition.^28^

Of the two studies that reported a significant difference favouring the control group, one had fair methodological quality and compared RCCT to usual care,^25^ while the other had good methodological quality and compared RCCT to leisure-based activities.^30^

Of the remaining five studies that did not find statistically significant differences between groups, two studies, one with good,^24^ and another with excellent methodological quality compared RCCT to leisure-based activities,^34^ one with good methodological quality compared RCCT against usual care^29^ and two others, also with good methodological quality compared it to no intervention^22^ and finally, one with fair methodological quality^31^ did not report the condition of the control group.

### Follow-ups

Follow-ups ranged from 12 to 52 weeks. As reported previously, of the 16 studies included in this systematic review, 8 reported a statistically significant improvement in the RCCT group at post-intervention. However, only one of these eight studies maintained a statistically significance favouring the RCCT group at follow-up.^35^

Conversely, one study that showed no significant differences between groups at post-intervention reported a statistically significant difference at the 12-week follow-up.^23^

## Discussion

This systematic review provides an overview of the current evidence regarding the effectiveness of RCCT in older adults with MCI. The results were unclear regarding the effectiveness of RCCT. When RCCT was compared to leisure-based activities (8 studies), statistically significant differences favouring the RCCT group were found in 11 out of 25 cognitive function assessments, when the comparator was usual care (5 studies), differences were observed in 9 out of 16 assessments, and when compared to no intervention or not reported (4 studies), differences were found in 6 out of the 8 assessments. The conflicting findings highlight the need for further studies. The methodological quality of the included studies varied, with most achieving good quality scores on the PEDro scale.

**Figure 2.**
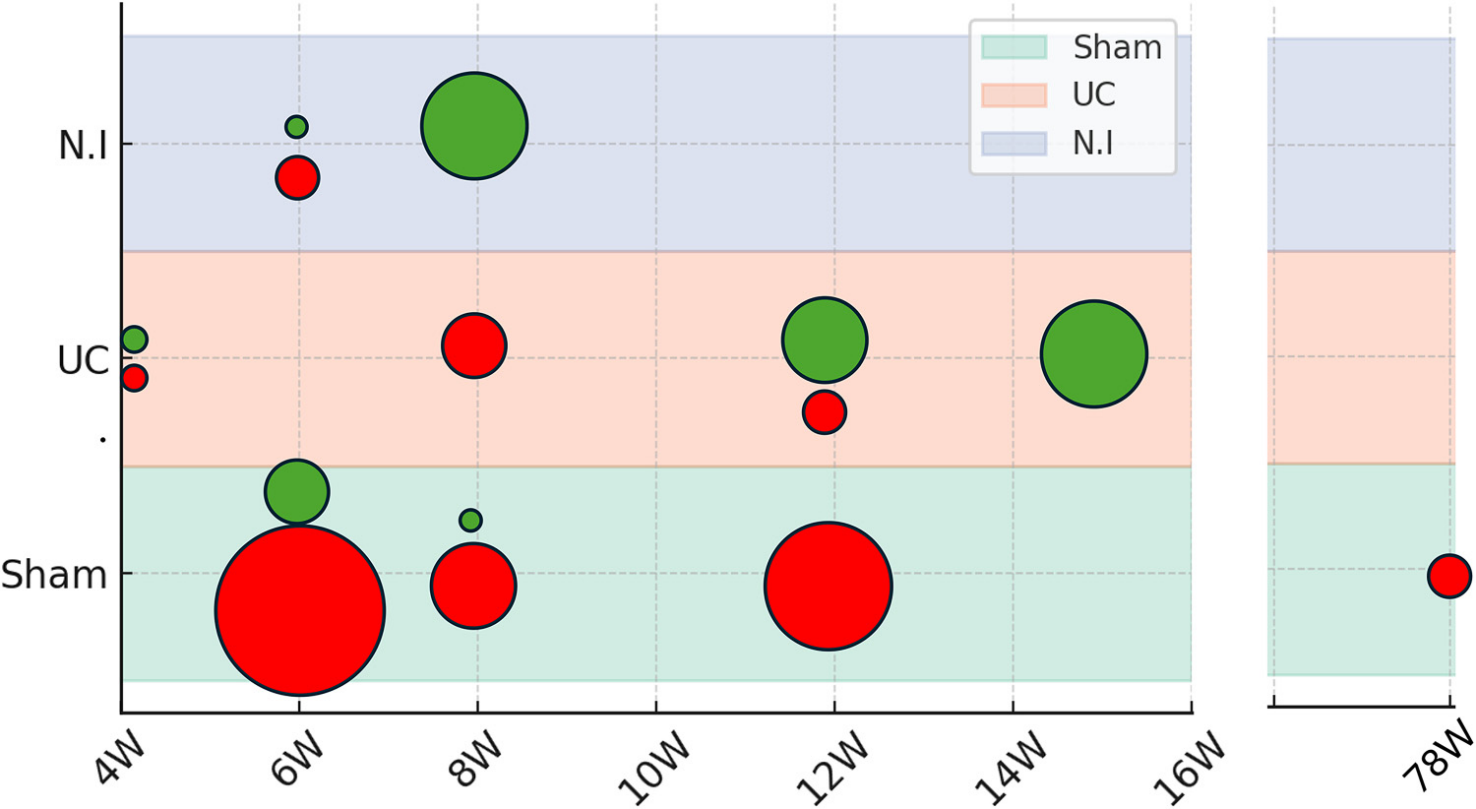
Results by comparator and intervention duration (in weeks) across all cognitive domains.

The diagram of Figure 2 visually summarizes the results by comparator (no intervention – NI, usual care – UC, and leisure-based activities – Sham) and intervention duration (in weeks) across all cognitive domains. Studies that reported statistically significant differences favouring the RCCT are shown in green, with the size of the green circles proportional to the number of cognitive assessments in which significant effects were observed. In contrast, studies with no statistically significant differences, or with differences favouring the control group, are represented in red, with circle size reflecting the number of assessments without significant differences.

A significant challenge in synthesizing the findings of the included studies was the high heterogeneity in the technologies employed and the cognitive training they facilitate. The studies used a wide range of digital solutions, from web-based applications like BrainHQ to virtual reality platforms like VRRS and tablet-based solutions such as Neuro-World. Besides, the digital solutions used are highly diverse, each has unique approaches and tasks for enhancing specific cognitive domains, and do not always target all (or the same) cognitive domains. Even studies that used the same digital solution and trained the same cognitive domains employed a diversity of training tasks. Similarly, the assessment instruments used across studies varied widely, even when the study authors considered to be assessing the same cognitive domain. Memory, for instance, was measured using 29 different instruments, highlighting substantial variability that makes comparisons difficult and raises concerns about whether studies are truly measuring the same construct.

In some studies, and despite using the same instrument, different authors claim to be assessing different cognitive domains. For example, the Trail Making Test B was reported as evaluating attention in one study^29^ and executive functions in another study,^23^ leading to potential misinterpretation of results. Additionally, the variation in control group conditions further difficult the interpretation of outcomes. Given these inconsistencies, reaching a consensus on which cognitive domains to assess and which instruments to use would be highly beneficial. Without agreement on what to measure and how to measure it, comparisons across studies become tenuous and conclusions uncertain. Standardizing outcome measures across studies would improve comparability, enhance the quality of evidence, and support more robust conclusions about the efficacy of RCCT. Previous systematic reviews on non-RCCT,^10,11,37^ also showed methodological variability among studies and consistently highlighted it as a limitation.^10,11,37^

Other important questions emerged from the results, which require investigation in future studies. One key issue is whether leisure-based activities, such as playing Sudoku or crossword puzzles, impact cognitive function. Two studies^30,36^ with good methodological quality found statistically significant improvements in memory^30,36^ and global cognition^30,36^ in favour of the leisure-based activities when compared to RCCT, highlighting the need for clear and detailed reporting of the tasks involved in such interventions.

With respect to usual care, although its content was heterogeneous and not consistently documented across studies, some studies explicitly reported the inclusion of conventional face-to-face cognitive interventions (e.g. see reference^29^). Studies that found no statistically significant differences between RCCT and face-to-face training should not be interpreted as evidence of ineffectiveness. On the contrary, these findings suggest that RCCT may offer comparable cognitive benefits while providing a more accessible, flexible, and potentially cost-effective alternative to in-person programs.

Another important consideration is the duration of the intervention. It remains unclear whether longer or more intensive RCCT programs result in better cognitive outcomes. As this review was inconclusive, further research is needed to determine whether extended interventions consistently lead to superior cognitive benefits.

Furthermore, does the impact of the intervention vary depending on the cognitive domain targeted? Or are certain cognitive domains more frequently trained and assessed? Previous reviews on CCT (not remote) conducted in individuals with subjective cognitive decline and MCI demonstrated varying benefits for different domains; for instance, most reviews reported memory-related benefits.^10,11^

The variation in outcomes may be attributable not only to differences in intervention duration and intensity but also to the nature of the intervention itself, for instance, a task designed to train memory may also train language processing.

### Limitations

The included studies exhibited considerable variability in several aspects, which limited the ability to synthesize findings quantitatively. A major challenge was the high heterogeneity of the technologies used, with studies employing a wide range of digital solutions and, even within the same platform, diverse training tasks. Control interventions also differed substantially, making comparisons difficult. Furthermore, decisions had to be made regarding which cognitive domain was primarily assessed by each instrument when multiple domains were reported; this was addressed through consultation with a panel of neuropsychology experts. Intervention duration and intensity varied widely, from short programs with few sessions to longer, more intensive interventions, which may have influenced outcomes. Collectively, these factors, along with the limited number of studies per cognitive domain and the lack of common comparators, prevented the planned meta-analysis.

## Conclusion

The findings of this review highlight important implications for both clinical practice and future research. From a clinical perspective, RCCT shows promise as a scalable and accessible approach to support cognitive health, particularly in settings with limited access to traditional services. However, its effective integration into practice requires more robust evidence of its effectiveness. Greater methodological consistency is essential, including the standardization of cognitive domains assessed, the use of validated and domain-specific instruments, and clear reporting of control group conditions to enable more reliable comparisons and stronger conclusions. Future research should prioritize methodological consistency by adopting standardized intervention protocols, comparator conditions, and validated outcome measures aligned with specific cognitive domains to determine the long-term impact and clinical relevance of RCCT. Future research would also benefit from the adoption of reporting guidelines that require explicit definition of trained cognitive domains and comprehensive documentation of training tasks, enabling more robust cross-study comparisons in RCCT research.

## Supplemental Material

sj-docx-1-dhj-10.1177_20552076261421682 - Supplemental material for The effectiveness of remote computerized cognitive training for older adults with mild cognitive impairment: A systematic review Supplemental material, sj-docx-1-dhj-10.1177_20552076261421682 for The effectiveness of remote computerized cognitive training for older adults with mild cognitive impairment: A systematic review by Ana Isabel Martins, Anabela G. Silva, Joana Pais and Nelson P. Rocha in DIGITAL HEALTH

sj-docx-2-dhj-10.1177_20552076261421682 - Supplemental material for The effectiveness of remote computerized cognitive training for older adults with mild cognitive impairment: A systematic review Supplemental material, sj-docx-2-dhj-10.1177_20552076261421682 for The effectiveness of remote computerized cognitive training for older adults with mild cognitive impairment: A systematic review by Ana Isabel Martins, Anabela G. Silva, Joana Pais and Nelson P. Rocha in DIGITAL HEALTH

## Appendix

**Table 1.** Summary of the main results per cognitive function.

	Study	Measurement	Baseline mean (SD)	Post-intervention	Follow-up 1	Follow-up 2	Statistical differences between groups
Global Cognition	Barnes et al. 2008^20^	Repeatable Battery for the Assessment of Neuropsychological Status (RBANS)	EG: 85.2 (11.5) CG: 87.8 (13.6)	EG: 0.36 (−0.07; 0.80)* CG: 0.03 (−0.39-0.45)*	NA	NA	No
	Rosen et al. 2011^21^	Repeatable Battery for the Assessment of Neuropsychological Status (RBANS)	EG: 443.83 (33.1) CG: 477.33 (48.45)	Not reported	NA	NA	No
	Han et al. 2017^27^	Mini-mental state examination (MMSE)	EG: 25.49 (3.40) CG: 25.83 (2.92)	EG: 26.37 (2.99) CG: 25.76 (3.28)	NA	NA	No
	Li et al. 2019^28^	Mini-mental state examination (MMSE)	EG: 28.0 (1.7) CG: 28.0 (1.7)	EG: 0.23 (0.01 to 0.44)* CG: −0.50 (−0.72 to −0.27*	Not reported	NA	Yes (favouring the RCCT group)
	Baik et al. 2023^33^	Montreal Cognitive Assessment (MoCA)	EG: 21.12 (1.27) CG: 20.84 (1.46)	EG: 23.84 (2.21) CG: 20.52 (1.76)	NA	NA	Yes (favouring the RCCT group)
	Duff et al. 2021^30^	Repeatable Battery for the Assessment of Neuropsychological Status (RBANS)	EG: 85.1 (13.5) CG: 85.7 (14.2)	EG: 85.6 (14.6) CG: 89.1(13.5)	EG: 86.1(14.9)^Ω^ CG: 87.5 (14.0)^Ω^	NA	No
	Brill et al. 2024^34^	Montreal Cognitive Assessment (MoCA)	EG: 27.2(2.24) Active CG: 26.7(3.28) Waitlist control: 26.9 (2.68)	Not reported	NA	NA	No
	Han et al. 2024^35^	Montreal Cognitive Assessment (MoCA)	EG: 17.32 (2.69) CG: 16.85 (2.26)	EG: 19.96 (2.57) CG: Not reported	Not reported	NA	Yes (favouring the RCCT group)
		Mini-mental state examination (MMSE)	EG: 21.16 (2.82) CG: 20.93 (2.40)	EG: 19.96 (2.57) CG: Not reported	Not reported	NA	Yes (favouring the RCCT group)
	Devanand et al. 2022^36^	11-item Alzheimer's Disease Assessment Scale Cognitive (ADAS-Cog)	EG: 9.5 (–3.5) CG: 9.6 (–3.5)	EG: 9.93(0.81)** CG: 8.61 (0.62)**	NA	NA	Yes (favouring the control group)
		Neuropsychological composite	EG: -0.1 (1.0) CG: 0.1 (1.0)	EG: -0.24 (0.09) -0.28 (0.09)	NA	NA	No
Attention	Barnes et al. 2008^20^	RBANS attention subscale	EG: 93.3 (14.0) CG: 95.3 (16.0)	EG: -0.11 (-0.50; 0.28)* CG: -0.15 (-0.61; 0.31)*	NA	NA	No
	Rosen et al. 2011^21^	RBANS Digit span (forward)	EG: 10.00 (2.28) CG: 11.50 (2.88)	Not reported	NA	NA	No
		RBANS Coding	EG: 36.33 (4.13) CG: 34.17 (5.42)	Not reported	NA	NA	No
	Finn et al. 2017^22^	Rapid visual information processing (RVP)	EG: 0.87 (0.07) CG: 0.85 (0.09)	EG: 0.90 (0.05) CG: 0.79 (0.13)	NA	NA	Yes (favouring the RCCT group)
	Wu et al. 2017^25^	CNS Vital signs subtest	EG: 100.18 (12.97)◊ CG: 96.25 (12.94)◊	EG: 99.44 (12.78)◊ CG: 100.65 (13.01)◊	EG: 103.11 (12.42)ϐ◊ CG: 99.29 (12.89)ϐ◊	NA	No
	Nousia et al. 2021^27^	Trail Making Test A	EG: 98.44 (27.31) CG: 110.14 (37.02)	EG: 80.72 (23.45) CG: 113.67 (37.36)	NA	NA	Yes (favouring the RCCT group)
	Li et al. 2019^28^	Chinese version of Addenbrooke's cognitive examination-revised (ACE-R) - Attention	EG: 17.1 (1.1) CG: 17.2 (1.0)	EG: 0.17 (−0.03 to 0.37)* CG: −0.48 (−0.74 to −0.23)*	Not reported	NA	Yes (favouring the RCCT group)
	Manenti et al. 2020^29^	Trail Making Test A	EG: 58.4 (29.0) CG: 46.8 (16.0)	EG: 52.0 (25.3) CG: 47.1 (16.2)	EG: 58.1 (24.7)ϐ CG: 52.4 (21.4)ϐ	EG: 55.3 (24.1)Δ CG: 60.0 (37.9)Δ	No
		Trail Making Test B	EG: 251.3 (146.3) CG: 206.9 (123.8)	EG: 216.6 (140.0) CG: 231.2 (184.7)	EG: 219.1 (139.4)ϐ CG: 219.8 (164.3)ϐ	EG: 245.3 (126.4)Δ CG: 268.2 (201.5)Δ	No
	Baik et al.2023^33^	Digit Span Test (DST)	EG: 10.48 (2.58) CG: 10.12 (2.65)	EG: 13.44 (2.68) CG: 10.88 (3.63	NA	NA	Yes (favouring the RCCT group)
Executive function	Barnes et al. 2008^20^	Design fluency tests from the Delis-Kaplan Executive Function Scale	EG: 5.0 (3.0) CG: 4.0 (2.4)	EG: 0.08 (-0.3; 0.49)* CG: 0.19 (-0.26; 0.63)*	NA	NA	No
		California trail making test	EG: 137.0 (51.2) CG: 149.0 (58.6)	EG: -0.11 (-0.56; 0.35)* CG: -0.08 (-0.49; 0.33)*	NA	NA	No
	Finn et al. 2017^22^	Intra-/extra-dimensional set shifting (IED)	EG: 31.13 (48.70) CG: 100.25 (78.79)	EG: 22.25 (22.11) CG: 94.13 (78.61)	NA	NA	No
	Hyer et al. 2016^23^	Trail Making Test B	EG: 132.38 (47.92) CG: 133.97 (41.56)	EG: 118.92 (43.49) CG: 112.57 (39.74)	EG: 102.92 (32.98)φ CG: 112.87 (32.15)φ	NA	No
	Li et al. 2019^28^	Stroop Color-Word Test (SCWT)	EG: 77.2 (21.8) CG: 84.7 (29.3)	EG: −0.09 (−0.33 to 0.15)* CG: -0.26 (-0.6 to 0.07)*	Not reported	NA	No
	Lin et al. 2021^31^	EXAMINER	Not reported	Not reported	Not reported	Not reported	No
	Baik et al. 2023^33^	Phonemic Word Fluency Test (PWF)	EG: 14.68 (± 5.10) CG: 15.24 (4.82)	EG: 17,72 (4,64) CG: 15.72 (4.26)	NA	NA	Yes (favouring the RCCT group)
Language	Barnes et al. 2008^20^	RBANS language	EG: 91.5 (10.3) CG: 93.6 (12.1)	EG: 0.30 (-0.13; 0.74)* CG: 0.29 (-0.14; 0.72)*	NA	NA	No
		Boston Naming Test (BNT)	EG: 26.4 (3.8) CG: 26.8 (2.4)	EG: -0.05 (-0.51; 0.42)* CG: 0.19 (-0.21; 0.59)*	NA	NA	No
		Controlled Oral Word Association Test (COWAT)	EG: 35.0 (13.7) CG: 40.2 (16.0)	EG: -0.20 (-0.68; 0.28)* CG: 0.02 (-0.37; 0.41)*	NA	NA	No
	Rosen et al. 2011^21^	RBANS Picture naming	EG: 9.83 (0.41) CG: 9.83 (0.41)	EG: Not reported CG: Not reported	NA	NA	No
		RBANS Semantic fluency	EG: 15.83 (3.76) CG: 15.50 (5.05)	EG: Not reported CG: Not reported	NA	NA	No
	Lin et al. 2016^24^	Examiner verbal fluency	EG: 0.55 (0.48) CG: 0.34 (0.69)	EG: 0.50 (0.57) CG: 0.21 (0.70)	NA	NA	No
	Nousia et al. 2021^27^	Boston Naming Test (BNT)	EG: 13.56 (1.45) CG: 13.10 (1.64)	EG: 14.60 (0.65) CG: 12.90 (2.63)	NA	NA	Yes (favouring the RCCT group)
		Semantic Fluency measure (SF)	EG: 30.44 (7.76) CG: 38.05 (7.49)	EG: 40.60 (7.17) CG: 34.90 (5.54)	NA	NA	
	Li et al. 2019^28^	Chinese version of Addenbrooke's cognitive examination-revised (ACE-R) - Language	EG: 24.5 (1.7) CG: 23.2 (1.9)	EG: 0.01 (−0.16 to 0.18)* CG: −0.05 (−0.37 to 0.26)*	Not reported	NA	No
	Manenti et al. 2020^29^	Verbal fluency, phonemic – FPL	EG: 29.7 (7.1) CG: 28.9 (8.4)	EG: 31.5 (9.1 CG: 31.7 (8.8)	EG: 29.2 (6.6)ϐ CG: 31.2 (11.5)ϐ	EG: 30.1 (7.6)Δ CG: 30.4 (7.9)Δ	No
		Verbal fluency, semantic - FPC	EG: 27.8 (5.8) CG: 30.9 (6.3)	EG: 30.8 (6.8) CG: 29.4 (6.0)	EG: 30.1 (5.5)ϐ CG: 29.1 (7.8)ϐ	EG: 29.1 (6.4)Δ CG: 29.2 (6.1)Δ	Yes (favouring the RCCT group)
	Baik et al.2023^33^	Semantic Word Fluency Test (SWF)	EG: 19.36 (4.16) CG: 19.72 (3.32)	EG: 22.68 (3,82) CG: 19.40 (3.43)	NA	NA	Yes (favouring the RCCT group)
	Brill et al.2024^34^	Graded Naming Task (GNT-30)	Not reported	Not reported	NA	NA	No
Visuo-constructional function	Barnes et al. 2008^20^	RBANS Figure copy	EG: 103.4 (14.3) CG: 103.4 (14.6)	EG: -0.07 (-0.39; 0.26)* CG: 0.44 (-0.03; 0.92)*	NA	NA	No
	Rosen et al. 2011^21^	RBANS Figure copy	EG: 19.17 (0.75) CG: 17.83 (2.48)	EG: Not reported CG: Not reported	NA	NA	No
		RBANS Line orientation	EG: 17.17 (2.48) CG: 17.50 (1.87)	EG: Not reported CG: Not reported	NA	NA	No
	Nousia et al. 2021^27^	Clock Drawing Test (CDT)	EG: 13.68 (1.25) CG: 14.0 (1.34)	EG: 14.44 (0.82) CG: 13.90 (1.18)	NA	NA	Yes (favouring the RCCT group)
	Li et al. 2019^28^	Chinese version of Addenbrooke's cognitive examination-revised (ACE-R) - Visuospatial ability	EG: 15.5 (0.9) CG: 14.9 (1.2)	EG: −0.05 (−0.27 to 0.17)* CG: 0.12 (−0.11 to 0.35)*	Not reported	NA	No
		The Rey-Osterrieth complex figure test (ROCFT)	EG: 34.1 (4.6) CG: 34.3 (2.6)	EG: 0.13 (−0.13 to 0.39)* CG: −0.25 (−0.49 to −0.01)*	Not reported	NA	Yes (favouring the RCCT group)
	Manenti et al. 2020^29^	Clock Drawing Test (CDT)	EG: 2.0 (0.8) CG: 1.9 (0.7)	EG: 1.7 (0.8) CG: 1.8 (0.7)	EG: 1.7 (0.9)ϐ CG: 2.0 (1.1)ϐ	EG: 1.7 (0.8)Δ CG: 1.8 (0.7)Δ	Yes (favouring the RCCT group)
	Brill et al. 2024^34^	Rey-Osterrieth Complex Figure Test (ROCFT)	Not reported	Not reported	NA	NA	No
Processing Speed	Lin et al. 2016^24^	Useful Field of View (UFOV)	EG: 136.35 (87.42) CG: 96.63 (48.67)	EG: 63.96 (22.22) CG: 87.65 (59.53)	NA	NA	Yes (favouring the RCCT group)
	Wu et al. 2017^25^	CNS Vital signs subtest	EG: 104.32 (14.75)◊ CG: 95.34 (14.67)◊	EG: 108.61 (13.74)◊ CG: 101.55 (14.67)◊	EG: 109.25 (13.56)ϐ◊ CG: 103.17 (14.91)ϐ◊	NA	No
	Nousia et al. 2021^27^	Trail Making Test B	EG: 222.48 (53.79) CG: 238.38 (52.25)	EG: 174.16 (37.11) CG: 237.86 (43.73)	NA	NA	Yes (favouring the RCCT group)
	Li et al. 2019^28^	Symbol digit substitution test (SDS)	EG: 40.1(10.9) CG: 36.3(11.0)	EG: 0.07(−0.16to0.30)* CG: 0.13(−0.11to 0.38)*	Not reported	NA	No
	Lin et al.2020^32^	Useful Field of View (UFOV)	EG: 5.89 (0.51) CG: 5.89 (0.50)	Not reported	Not reported	NA	Yes (favouring the RCCT group)
Memory	Barnes et al. 2008^20^	RBANS delayed memory	EG: 71.4 (19.2) CG: 74.6 (21.4)	EG: 0.40 (-0.11; 0.90)* CG: -0.13 (-0.47; 0.20)*	NA	NA	No
		California Verbal Learning Test - II (CVLT-II total learned)	EG: 35.3 (11.5) CG: 33.9 (13.7)	EG: -0.08 (-0.42; 0.26)* CG: -0.24 (-0.72; 0.25)*	NA	NA	No
		CVLT delayed free recall	EG: 5.1 (3.9) CG: 5.3 (4.3)	EG: 0.07 (-0.32; 0.46)* CG: -0.19 (-0.64; 0.26)*	NA	NA	No
		RBANS immediate memory	EG: 84.8 (12.6) CG: 85.7 (18.4)	EG: 0.32 (-0.18; 0.83)* CG: -0.05 (-0.40; 0.30)*	NA	NA	No
		Spatial span test	EG: 13.0 (3.5) CG: 12.2 (1.9)	EG: 0.53 (0.02; 1.03)* CG: -0.32 (-0.59; -0.05)*	NA	NA	Yes (favouring the RCCT group)
	Rosen et al. 2011^21^	RBANS Story recall	EG: 4.67 (3.08) CG: 7.17 (3.25)	EG: Not reported CG: Not reported	NA	NA	Yes (favouring the RCCT group)
		RBANS List recall	EG: 1.33 (1.21) CG: 1.17 (2.86)	EG: Not reported CG: Not reported	NA	NA	Yes (favouring the RCCT group)
		RBANS List recognition	EG: 16.33 (1.37) CG: 16.00 (2.45)	EG: Not reported CG: Not reported	NA	NA	Yes (favouring the RCCT group)
	Finn et al. 2017^22^	Pattern recognition memory (PRM)	EG: 89.06 (11.57) CG: 76.04 (15.05)	EG: 90.62 (9.39) CG: 80.22 (15.39)	NA	NA	No
	Hyer et al. 2016^23^	Wechsler Memory Scale (Span Board subtest)	EG: 8.79 (2.48) CG: 9.73 (3.10)	EG: 11.54 (3.37) CG: 10.77 (3.07)	EG: 12.13 (3.46)φ CG: 10.63 (3.12)φ	NA	Yes (favouring the RCCT group)
		Wechsler Memory Scale (Letter Number Sequencing subtest)	EG: 9.63 (3.13) CG: 10.00 (2.85)	EG: 10.90 (2.38) CG: 10.53 (2.46)	EG: 10.83 (3.02)φ CG: 10.37 (2.50)φ	NA	No
	Lin et al. 2016^24^	Examiner	EG: −0.58 (0.71) CG: 0.26 (0.68)	EG: 0.11 (0.37) CG: −0.06 (0.76)	NA	NA	No
	Wu et al. 2017^25^	CNS Vital signs subtest	EG: 98.65 (13.53)◊ CG: 93.91 (13.53)◊	EG: 96.62 (13.12)◊ CG: 103.11 (13.53)◊	EG: 101.58 (12.98)ϐ◊ CG: 98.91 (13.53)ϐ◊	NA	Yes (favouring the control group)
	Han et al. 2017^26^	Word List Recognition Test (WLRT)	EG: 4.63 (2.31) CG: 5.14 (2.35)	EG: 5.74 (2.26) CG: 5.50 (2.19)	NA	NA	Yes (favouring the RCCT group)
	Nousia et al. 2021^27^	Word recognition	EG: 18.96 (1.43) CG: 19.24 (1.09)	EG: 19.68 (0.48) CG: 19.48 (0.81)	NA	NA	Yes (favouring the RCCT group)
		Delayed memory test	EG: 1.80 (0.76) CG: 1.43 (1.29)	EG: 3.04 (1.21) CG: 0.67 (0.58)	NA	NA	Yes (favouring the RCCT group)
		Digital span backward (DSB)	EG: 4.48 (1.23) CG: 4.52 (1.29)	EG: 4.64 (1.08) CG: 4.00 (1.30)	NA	NA	No
	Li et al. 2019^28^	Chinese version of Addenbrooke's cognitive examination-revised (ACER)	EG: 22.3 (4.5) CG: 23.0 (2.6)	EG: -0.34 (0.11 to −0.58)* CG: −0.35 (−0.55 to −0.15)*	Not reported	NA	Yes (favouring the RCCT group)
		The auditory verbal learning test (AVLT)	EG: 20.3 (5.0) CG: 16.9 (6.6)	EG: 0.07 (−0.15 to 0.29)* CG: 0.23 (−0.03 to 0.48)*	Not reported	NA	No
	Manenti et al. 2020^29^	Auditory Verbal Learning Test (AVLT) delayed recall	EG: 4.0 (3.1) CG: 4.5 (3.2)	EG: 4.3 (3.3) CG: 4.2 (3.0)	EG: 3.9 (3.9)ϐ CG: 4.2 (2.6)ϐ	EG: 3.6 (3.3)Δ CG: 4.8 (3.9)Δ	No
		Free and Cued Selective Reminding Test (FCSRT) delayed total recall	EG: 10.4 (2.1) CG: 10.8 (2.1)	EG: 10.3 (2.5) CG: 10.9 (1.9)	EG: 10.4 (1.8)ϐ CG: 10.8 (1.5)ϐ	EG: 10.4 (2.0)Δ CG: 11.2 (1.2)Δ	No
		Auditory Verbal Learning Test (AVLT) immediate recall	EG: 29.2 (6.8) CG: 30.2 (7.7)	EG: 29.4 (7.4) CG: 30.1 (6.5)	EG: 29.9 (8.6)ϐ CG: 30.9 (6.6)ϐ	EG: 26.1 (7.6)Δ CG: 31.5 (7.8)Δ	No
		Free and Cued Selective Reminding Test (FCSRT) immediate total recall	EG: 32.0 (4.3) CG: 33.4 (3.2)	EG: 32.1 (5.5 CG: 32.5 (4.9)	EG: 31.2 (6.5)ϐ CG: 33.1 (3.9)ϐ	EG: 32.0 (4.4)Δ CG: 33.5 (3.6)Δ	No
	Duff et al.2021^30^	RBANS Auditory Memory/Attention Index	EG: 88.1 (12.5) CG: 90.8 (12.9)	EG: 89.0 (13.1) CG: 94.3 (12.1)	EG: 87.7 (14.8)Ω CG: 90.6 (12.7)Ω	NA	Yes (favouring the control group)
	Lin et al. 2021^31^	Brief Visuospatial Memory Test (BVMT)-R	Not reported	Not reported	Not reported	Not reported	No
	Baik et al.2023^33^	Verbal Learning Test (VLT)	EG: 15.32 (4.46) CG: 15.04 (4.96)	EG: 16,88 (4.00) CG: 15.20 (5.27)	NA	NA	Yes (favouring the RCCT group)
		Digit Span Tests (DST)	EG: 10.48 (2.58) CG: 10.12 (2.65)	EG: 13.44 (2.68) CG: 10.88 (3.63)	NA	NA	Yes (favouring the RCCT group)
	Brill et al.2024^34^	Auditory Verbal Learning Test (AVLT) + Digit span test	Not reported	Not reported	NA	NA	No
	Han et al. 2024^35^	Prospective and Retrospective Memory Questionnaire (PRMQ)	EG: 39.35 (7.53) CG: 39.02 (6.96)	Not reported	Not reported		Yes (favouring the RCCT group)

* Mean change for within-group differences (95% confidence interval).

** Mean (SE).

◊ Values in least square means.

Ω 1 year.

ϐ 16 weeks.

Δ 7 months.

φ 3 months.

RCCT: remote computerized cognitive training.

**Table 2.** Methodological quality of included studies (1. Eligibility criteria; 2. Random allocation; 3. Concealment; 4. Groups similar at baseline; 5. Blinding of subjects; 6. Blinding of who administered the intervention; 7. Blinding of assessors; 8. Measures of outcome for 85% of subjects; 9. Treatment or control as allocated; 10. Statistics for between-group comparisons; 11. Point and variability measures.).

Authors (year)	1	2	3	4	5	6	7	8	9	10	11	Total (2–11)*
Barnes et al. 2008^20^	1	1	1	1	1	0	1	1	1	1	1	9
Rosen et al. 2011^21^	1	1	1	1	1	1	1	1	1	1	1	10
Finn et al. 2017^22^	1	1	1	1	0	0	1	0	1	1	1	7
Hyer et al. 2016^23^	1	1	0	1	1	0	1	1	0	1	1	7
Lin et al. 2016^24^	1	1	0	1	0	0	1	1	1	1	1	7
Wu et al. 2017^25^	1	1	0	1	0	0	0	0	0	1	1	4
Han et al. 2017^26^	1	1	1	1	0	0	1	0	1	1	1	7
Nousia et al. 2021^27^	1	1	0	1	0	0	1	1	1	1	1	7
Li et al. 2019^28^	1	1	0	0	0	0	1	0	0	1	1	4
Manenti et al. 2020^29^	1	1	1	1	0	0	1	1	1	1	1	8
Duff et al. 2021^30^	1	1	1	1	1	0	1	0	0	1	1	7
Lin et al. 2021^31^	1	1	1	1	1	0	1	1	1	1	0	8
Lin et al. 2020^32^	1	1	0	1	0	0	1	1	1	1	1	7
Baik et al. 2023^33^	1	1	0	1	0	0	1	1	1	1	1	7
Brill et al. 2024^34^	1	1	1	1	1	1	1	1	1	1	1	10
Han et al. 2024^35^	1	1	1	1	0	0	1	1	1	1	1	8
Devanand et al. 2022^36^	1	1	0	1	0	0	1	1	1	1	1	7

Legend: 0 – No; 1 – Yes: * first item of the scale not included in the total score.

**Table 3.** Full search strategy for PubMed.

**Database:** PubMed
**Date of search:** 7 January 2025
**Search string:** ((“Mild Cognitive Impairment"[MeSH Terms] OR “Mild Cognitive Impairment"[Title/Abstract] OR MCI[Title/Abstract]) AND (“Cognitive Training"[MeSH Terms] OR “Cognitive Rehabilitation"[MeSH Terms] OR “Cognitive training"[Title/Abstract] OR “Cognitive rehabilitation"[Title/Abstract] OR “Computerized cognitive training"[Title/Abstract] OR “Digital cognitive training"[Title/Abstract] OR “Virtual reality"[Title/Abstract] OR “Serious games"[Title/Abstract] OR “Web-based"[Title/Abstract] OR “Mobile"[Title/Abstract]) AND (“Randomized Controlled Trial"[Publication Type] OR “randomized controlled trial"[Title/Abstract] OR RCT[Title/Abstract]))
**Filters applied:** • Humans • Age: 60 years and older
Article type: Exclude reviews, systematic reviews, and books • Language: English
