# Supplementary material for: The effectiveness of remote computerized cognitive training for older adults with mild cognitive impairment: A systematic review
Source: Digit Health. 2026 Feb 11;12:20552076261421682. doi: 10.1177/20552076261421682 (PMC12901901; doi:10.1177/20552076261421682)
Supplement: sj-docx-1-dhj-10.1177_20552076261421682 - Supplemental material for The effectiveness of remote computerized cognitive training for older adults with mild cognitive impairment: A systematic review [file sj-docx-1-dhj-10.1177_20552076261421682.docx]

Table 1

|  | Study | Measurement | Baseline  Mean (SD) | Post-intervention | Follow-up 1 | Follow-up 2 | Statistical differences between groups |
| --- | --- | --- | --- | --- | --- | --- | --- |
| Global Cognition | Barnes, et al 2008 [20] | Repeatable Battery for the Assessment of Neuropsychological Status (RBANS) | EG: 85.2 (11.5)  CG: 87.8 (13.6) | EG: 0.36 (-0.07; 0.80)*  CG: 0.03 (-0.39-0.45)* | NA | NA | No |
|  | Rosen, *et al. 2011 [21]* | Repeatable Battery for the Assessment of Neuropsychological Status (RBANS) | EG: 443.83 (33.1)  CG: 477.33 (48.45) | Not reported | NA | NA | No |
|  | Han, et al. 2017 [27] | Mini mental state examination (MMSE) | EG: 25.49 (3.40)  CG: 25.83 (2.92) | EG: 26.37 (2.99)  CG: 25.76 (3.28) | NA | NA | No |
|  | Li *et al. 2019 [28]* | Mini mental state examination (MMSE) | EG: 28.0 (1.7)  CG: 28.0 (1.7) | EG: 0.23 (0.01 to 0.44)*  CG: −0.50 (−0.72 to −0.27* | Not reported | NA | Yes (favouring the RCCT group) |
|  | Baik, *et al.2023 [33]* | Montreal Cognitive Assessment (MoCA) | EG: 21.12 (1.27)  CG: 20.84 (1.46) | EG: 23.84 (2.21)  CG: 20.52 (1.76) | NA | NA | Yes (favouring the RCCT group) |
|  | Duff, *et al.2021 [30]* | Repeatable Battery for the Assessment of Neuropsychological Status (RBANS) | EG: 85.1 (13.5)  CG: 85.7 (14.2) | EG: 85.6 (14.6)  CG: 89.1(13.5) | EG: 86.1(14.9)^Ω^  CG: 87.5 (14.0)^Ω^ | NA | No |
|  | Brill, *et al.2024 [34]* | Montreal Cognitive Assessment (MoCA) | EG: 27.2(2.24)  Active CG: 26.7(3.28)  Waitlist control: 26.9 (2.68) | Not reported | NA | NA | No |
|  | Han, et al. 2024 [35] | Montreal Cognitive Assessment (MoCA) | EG: 17.32 (2.69) CG: 16.85 (2.26) | EG: 19.96 (2.57) CG: Not reported | Not reported | NA | Yes (favouring the RCCT group) |
|  |  | Mini mental state examination (MMSE) | EG: 21.16 (2.82) CG: 20.93 (2.40) | EG: 19.96 (2.57) CG: Not reported | Not reported | NA | Yes (favouring the RCCT group) |
|  | Devanand, et al. 2022 [36] | 11-item Alzheimer’s Disease Assessment Scale Cognitive (ADAS-Cog) | EG: 9.5 (–3.5)  CG: 9.6 (–3.5) | EG: 9.93(0.81)^**^  CG: 8.61 (0.62)^**^ | NA | NA | Yes (favouring the control group) |
|  |  | Neuropsychological composite | EG: -0.1 (1.0)  CG: 0.1 (1.0) | EG: -0.24 (0.09)  -0.28 (0.09) | NA | NA | No |
| Attention | Barnes, et al 2008 [20] | RBANS attention subscale | EG: 93.3 (14.0)  CG: 95.3 (16.0) | EG: -0.11 (-0.50; 0.28)*  CG: -0.15 (-0.61; 0.31)* | NA | NA | No |
|  | Rosen, *et al. 2011 [21]* | RBANS Digit span (forward) | EG: 10.00 (2.28)  CG: 11.50 (2.88) | Not reported | NA | NA | No |
|  |  | RBANS Coding | EG: 36.33 (4.13)  CG: 34.17 (5.42) | Not reported | NA | NA | No |
|  | Finn *et al. 2017 [22]* | Rapid visual information processing (RVP) | EG: 0.87 (0.07)  CG: 0.85 (0.09) | EG: 0.90 (0.05)  CG: 0.79 (0.13) | NA | NA | Yes (favouring the RCCT group) |
|  | Wu *et al. 2017 [25]* | CNS Vital signs subtest | EG: 100.18 (12.97)◊  CG: 96.25 (12.94)◊ | EG: 99.44 (12.78)◊  CG: 100.65 (13.01)◊ | EG: 103.11 (12.42)ϐ◊  CG: 99.29 (12.89)ϐ◊ | NA | No |
|  | Nousia, *et al. 2021 [27]* | Trail Making Test A | EG: 98.44 (27.31)  CG: 110.14 (37.02) | EG: 80.72 (23.45)  CG: 113.67 (37.36) | NA | NA | Yes (favouring the RCCT group) |
|  | Li *et al. 2019 [28]* | Chinese version of Addenbrooke's cognitive examination-revised (ACE-R) - Attention | EG: 17.1 (1.1)  CG: 17.2 (1.0) | EG: 0.17 (−0.03 to 0.37)*  CG: −0.48 (−0.74 to −0.23)* | Not reported | NA | Yes (favouring the RCCT group) |
|  | Manenti *et al. 2020 [29]* | Trail Making Test A | EG: 58.4 (29.0)  CG: 46.8 (16.0) | EG: 52.0 (25.3)  CG: 47.1 (16.2) | EG: 58.1 (24.7)ϐ  CG: 52.4 (21.4)ϐ | EG: 55.3 (24.1)Δ  CG: 60.0 (37.9)Δ | No |
|  |  | Trail Making Test B | EG: 251.3 (146.3)  CG: 206.9 (123.8) | EG: 216.6 (140.0)  CG: 231.2 (184.7) | EG: 219.1 (139.4)ϐ  CG: 219.8 (164.3)ϐ | EG: 245.3 (126.4)Δ  CG: 268.2 (201.5)Δ | No |
|  | Baik, *et al.2023 [33]* | Digit Span Test (DST) | EG: 10.48 (2.58)  CG: 10.12 (2.65) | EG: 13.44 (2.68)  CG: 10.88 (3.63 | NA | NA | Yes (favouring the RCCT group) |
| Executive function | Barnes, et al 2008 [20] | Design fluency tests from the Delis-Kaplan Executive Function Scale | EG: 5.0 (3.0)  CG: 4.0 (2.4) | EG: 0.08 (-0.3; 0.49)*  CG: 0.19 (-0.26; 0.63)* | NA | NA | No |
|  |  | California trail making test | EG: 137.0 (51.2)  CG: 149.0 (58.6) | EG: -0.11 (-0.56; 0.35)*  CG: -0.08 (-0.49; 0.33)* | NA | NA | No |
|  | Finn, *et al 2017 [22]* | Intra-/extra-dimensional set shifting (IED) | EG: 31.13 (48.70)  CG: 100.25 (78.79) | EG: 22.25 (22.11)  CG: 94.13 (78.61) | NA | NA | No |
|  | Hyer, *et al 2016 [23]* | Trail Making Test B | EG: 132.38 (47.92)  CG: 133.97 (41.56) | EG: 118.92 (43.49)  CG: 112.57 (39.74) | EG: 102.92 (32.98)φ  CG: 112.87 (32.15)φ | NA | No |
|  | Li *et al. 2019 [28]* | Stroop Color-Word Test (SCWT) | EG: 77.2 (21.8)  CG: 84.7 (29.3) | EG: −0.09 (−0.33 to 0.15)*  CG: -0.26 (-0.6 to 0.07)* | Not reported | NA | No |
|  | Lin *et al. 2021 [31]* | EXAMINER | Not reported | Not reported | Not reported | Not reported | No |
|  | Baik, *et al.2023 [33]* | Phonemic Word Fluency Test (PWF) | EG: 14.68 (± 5.10)  CG: 15.24 (4.82) | EG: 17,72 (4,64)  CG: 15.72 (4.26) | NA | NA | Yes (favouring the RCCT group) |
| Language | Barnes, et al 2008 [20] | RBANS language | EG: 91.5 (10.3)  CG: 93.6 (12.1) | EG: 0.30 (-0.13; 0.74)*  CG: 0.29 (-0.14; 0.72)* | NA | NA | No |
|  |  | Boston Naming Test (BNT) | EG: 26.4 (3.8)  CG: 26.8 (2.4) | EG: -0.05 (-0.51; 0.42)*  CG: 0.19 (-0.21; 0.59)* | NA | NA | No |
|  |  | Controlled Oral Word Association Test (COWAT) | EG: 35.0 (13.7)  CG: 40.2 (16.0) | EG: -0.20 (-0.68; 0.28)*  CG: 0.02 (-0.37; 0.41)* | NA | NA | No |
|  | Rosen, *et al. 2011 [21]* | RBANS Picture naming | EG: 9.83 (0.41)  CG: 9.83 (0.41) | EG: Not reported  CG: Not reported | NA | NA | No |
|  |  | RBANS Semantic fluency | EG: 15.83 (3.76)  CG: 15.50 (5.05) | EG: Not reported  CG: Not reported | NA | NA | No |
|  | Lin *et al. 2016 [24]* | Examiner verbal fluency | EG: 0.55 (0.48)  CG: 0.34 (0.69) | EG: 0.50 (0.57)  CG: 0.21 (0.70) | NA | NA | No |
|  | Nousia, *et al. 2021 [27]* | Boston Naming Test (BNT) | EG: 13.56 (1.45)  CG: 13.10 (1.64) | EG: 14.60 (0.65)  CG: 12.90 (2.63) | NA | NA | Yes (favouring the RCCT group) |
|  |  | Semantic Fluency measure (SF) | EG: 30.44 (7.76)  CG: 38.05 (7.49) | EG: 40.60 (7.17)  CG: 34.90 (5.54) | NA | NA |  |
|  | Li *et al. 2019 [28]* | Chinese version of Addenbrooke's cognitive examination-revised (ACE-R) - Language | EG: 24.5 (1.7)  CG: 23.2 (1.9) | EG: 0.01 (−0.16 to 0.18)*  CG: −0.05 (−0.37 to 0.26)* | Not reported | NA | No |
|  | Manenti *et al. 2020 [29]* | Verbal fluency, phonemic – FPL | EG: 29.7 (7.1)  CG: 28.9 (8.4) | EG: 31.5 (9.1  CG: 31.7 (8.8) | EG: 29.2 (6.6)ϐ  CG: 31.2 (11.5)ϐ | EG: 30.1 (7.6)Δ  CG: 30.4 (7.9)Δ | No |
|  |  | Verbal fluency, semantic - FPC | EG: 27.8 (5.8)  CG: 30.9 (6.3) | EG: 30.8 (6.8)  CG: 29.4 (6.0) | EG: 30.1 (5.5)ϐ  CG: 29.1 (7.8)ϐ | EG: 29.1 (6.4)Δ  CG: 29.2 (6.1)Δ | Yes (favouring the RCCT group) |
|  | Baik, *et al.2023 [33]* | Semantic Word Fluency Test (SWF) | EG: 19.36 (4.16)  CG: 19.72 (3.32) | EG: 22.68 (3,82)  CG: 19.40 (3.43) | NA | NA | Yes (favouring the RCCT group) |
|  | Brill, *et al.2024 [34]* | Graded Naming Task (GNT-30) | Not reported | Not reported | NA | NA | No |
| Visuo-constructional function | Barnes, et al 2008 [20] | RBANS Figure copy | EG: 103.4 (14.3)  CG: 103.4 (14.6) | EG: -0.07 (-0.39; 0.26)*  CG: 0.44 (-0.03; 0.92)* | NA | NA | No |
|  | Rosen, *et al. 2011 [21]* | RBANS Figure copy | EG: 19.17 (0.75)  CG: 17.83 (2.48) | EG: Not reported  CG: Not reported | NA | NA | No |
|  |  | RBANS Line orientation | EG: 17.17 (2.48)  CG: 17.50 (1.87) | EG: Not reported  CG: Not reported | NA | NA | No |
|  | Nousia, *et al. 2021 [27]* | Clock Drawing Test (CDT) | EG: 13.68 (1.25)  CG: 14.0 (1.34) | EG: 14.44 (0.82)  CG: 13.90 (1.18) | NA | NA | Yes (favouring the RCCT group) |
|  | Li *et al. 2019 [28]* | Chinese version of Addenbrooke's cognitive examination-revised (ACE-R) - Visuospatial ability | EG: 15.5 (0.9)  CG: 14.9 (1.2) | EG: −0.05 (−0.27 to 0.17)*  CG: 0.12 (−0.11 to 0.35)* | Not reported | NA | No |
|  |  | The Rey Osterrieth complex figure test (ROCFT) | EG: 34.1 (4.6)  CG: 34.3 (2.6) | EG: 0.13 (−0.13 to 0.39)*  CG: −0.25 (−0.49 to −0.01)* | Not reported | NA | Yes (favouring the RCCT group) |
|  | Manenti *et al. 2020 [29]* | Clock Drawing Test (CDT) | EG: 2.0 (0.8)  CG: 1.9 (0.7) | EG: 1.7 (0.8)  CG: 1.8 (0.7) | EG: 1.7 (0.9)ϐ  CG: 2.0 (1.1)ϐ | EG: 1.7 (0.8)Δ  CG: 1.8 (0.7)Δ | Yes (favouring the RCCT group) |
|  | Brill, *et al.2024 [34]* | Rey-Osterrieth Complex Figure Test (ROCFT) | Not reported | Not reported | NA | NA | No |
| Processing Speed | Lin *et al. 2016 [24]* | Useful Field of View (UFOV) | EG: 136.35 (87.42)  CG: 96.63 (48.67) | EG: 63.96 (22.22)  CG: 87.65 (59.53) | NA | NA | Yes (favouring the RCCT group) |
|  | Wu *et al. 2017 [25]* | CNS Vital signs subtest | EG: 104.32 (14.75)◊  CG: 95.34 (14.67)◊ | EG: 108.61 (13.74)◊  CG: 101.55 (14.67)◊ | EG: 109.25 (13.56)ϐ◊  CG: 103.17 (14.91)ϐ◊ | NA | No |
|  | Nousia, *et al. 2021 [27]* | Trail Making Test B | EG: 222.48 (53.79)  CG: 238.38 (52.25) | EG: 174.16 (37.11)  CG: 237.86 (43.73) | NA | NA | Yes (favouring the RCCT group) |
|  | Li *et al. 2019 [28]* | Symbol digit substitution test (SDS) | EG: 40.1(10.9)  CG: 36.3(11.0) | EG: 0.07(−0.16to0.30)*  CG: 0.13(−0.11to 0.38)* | Not reported | NA | No |
|  | Lin, *et al.2020 [32]* | Useful Field of View (UFOV) | EG: 5.89 (0.51)  CG: 5.89 (0.50) | Not reported | Not reported | NA | Yes (favouring the RCCT group) |
| Memory | Barnes, et al 2008 [20] | RBANS delayed memory | EG: 71.4 (19.2)  CG: 74.6 (21.4) | EG: 0.40 (-0.11; 0.90)*  CG: -0.13 (-0.47; 0.20)* | NA | NA | No |
|  |  | California Verbal Learning Test - II (CVLT-II total learned) | EG: 35.3 (11.5)  CG: 33.9 (13.7) | EG: -0.08 (-0.42; 0.26)*  CG: -0.24 (-0.72; 0.25)* | NA | NA | No |
|  |  | CVLT delayed free recall | EG: 5.1 (3.9)  CG: 5.3 (4.3) | EG: 0.07 (-0.32; 0.46)*  CG: -0.19 (-0.64; 0.26)* | NA | NA | No |
|  |  | RBANS immediate memory | EG: 84.8 (12.6)  CG: 85.7 (18.4) | EG: 0.32 (-0.18; 0.83)*  CG: -0.05 (-0.40; 0.30)* | NA | NA | No |
|  |  | Spatial span test | EG: 13.0 (3.5)  CG: 12.2 (1.9) | EG: 0.53 (0.02; 1.03)*  CG: -0.32 (-0.59; -0.05)* | NA | NA | Yes (favouring the RCCT group) |
|  | Rosen, *et al. 2011 [21]* | RBANS Story recall | EG: 4.67 (3.08)  CG: 7.17 (3.25) | EG: Not reported  CG: Not reported | NA | NA | Yes (favouring the RCCT group) |
|  |  | RBANS List recall | EG: 1.33 (1.21)  CG: 1.17 (2.86) | EG: Not reported  CG: Not reported | NA | NA | Yes (favouring the RCCT group) |
|  |  | RBANS List recognition | EG: 16.33 (1.37)  CG: 16.00 (2.45) | EG: Not reported  CG: Not reported | NA | NA | Yes (favouring the RCCT group) |
|  | Finn, *et al 2017 [22]* | Pattern recognition memory (PRM) | EG: 89.06 (11.57)  CG: 76.04 (15.05) | EG: 90.62 (9.39)  CG: 80.22 (15.39) | NA | NA | No |
|  | Hyer, *et al 2016 [23]* | Wechsler Memory Scale (Span Board subtest) | EG: 8.79 (2.48)  CG: 9.73 (3.10) | EG: 11.54 (3.37)  CG: 10.77 (3.07) | EG: 12.13 (3.46)φ CG: 10.63 (3.12)φ | NA | Yes (favouring the RCCT group) |
|  |  | Wechsler Memory Scale (Letter Number Sequencing subtest) | EG: 9.63 (3.13)  CG: 10.00 (2.85) | EG: 10.90 (2.38) CG: 10.53 (2.46) | EG: 10.83 (3.02)φ  CG: 10.37 (2.50)φ | NA | No |
|  | Lin *et al. 2016 [24]* | Examiner | EG: −0.58 (0.71)  CG: 0.26 (0.68) | EG: 0.11 (0.37)  CG: −0.06 (0.76) | NA | NA | No |
|  | Wu *et al. 2017 [25]* | CNS Vital signs subtest | EG: 98.65 (13.53)◊  CG: 93.91 (13.53)◊ | EG: 96.62 (13.12)◊  CG: 103.11 (13.53)◊ | EG: 101.58 (12.98)ϐ◊  CG: 98.91 (13.53)ϐ◊ | NA | Yes (favouring the control group) |
|  | Han, et al. 2017 [26] | Word List Recognition Test (WLRT) | EG: 4.63 (2.31)  CG: 5.14 (2.35) | EG: 5.74 (2.26)  CG: 5.50 (2.19) | NA | NA | Yes (favouring the RCCT group) |
|  | Nousia, *et al. 2021 [27]* | Word recognition | EG: 18.96 (1.43)  CG: 19.24 (1.09) | EG: 19.68 (0.48)  CG: 19.48 (0.81) | NA | NA | Yes (favouring the RCCT group) |
|  |  | Delayed memory test | EG: 1.80 (0.76) CG: 1.43 (1.29) | EG: 3.04 (1.21) CG: 0.67 (0.58) | NA | NA | Yes (favouring the RCCT group) |
|  |  | Digital span backward (DSB) | EG: 4.48 (1.23)  CG: 4.52 (1.29) | EG: 4.64 (1.08)  CG: 4.00 (1.30) | NA | NA | No |
|  | Li *et al. 2019 [28]* | Chinese version of Addenbrooke's cognitive examination-revised (ACER) | EG: 22.3 (4.5)  CG: 23.0 (2.6) | EG: -0.34 (0.11 to −0.58)*  CG: −0.35 (−0.55 to −0.15)* | Not reported | NA | Yes (favouring the RCCT group) |
|  |  | The auditory verbal learning test (AVLT) | EG: 20.3 (5.0)  CG: 16.9 (6.6) | EG: 0.07 (−0.15 to 0.29)*  CG: 0.23 (−0.03 to 0.48)* | Not reported | NA | No |
|  | Manenti *et al. 2020 [29]* | Auditory Verbal Learning Test (AVLT) delayed recall | EG: 4.0 (3.1)  CG: 4.5 (3.2) | EG: 4.3 (3.3)  CG: 4.2 (3.0) | EG: 3.9 (3.9)ϐ  CG: 4.2 (2.6)ϐ | EG: 3.6 (3.3)Δ  CG: 4.8 (3.9)Δ | No |
|  |  | Free and Cued Selective Reminding Test (FCSRT) delayed total recall | EG: 10.4 (2.1)  CG: 10.8 (2.1) | EG: 10.3 (2.5)  CG: 10.9 (1.9) | EG: 10.4 (1.8)ϐ  CG: 10.8 (1.5)ϐ | EG: 10.4 (2.0)Δ  CG: 11.2 (1.2)Δ | No |
|  |  | Auditory Verbal Learning Test (AVLT) immediate recall | EG: 29.2 (6.8)  CG: 30.2 (7.7) | EG: 29.4 (7.4)  CG: 30.1 (6.5) | EG: 29.9 (8.6)ϐ  CG: 30.9 (6.6)ϐ | EG: 26.1 (7.6)Δ  CG: 31.5 (7.8)Δ | No |
|  |  | Free and Cued Selective Reminding Test (FCSRT) immediate total recall | EG: 32.0 (4.3)  CG: 33.4 (3.2) | EG: 32.1 (5.5  CG: 32.5 (4.9) | EG: 31.2 (6.5)ϐ  CG: 33.1 (3.9)ϐ | EG: 32.0 (4.4)Δ  CG: 33.5 (3.6)Δ | No |
|  | Duff, *et al.2021 [30]* | RBANS Auditory Memory/Attention Index | EG: 88.1 (12.5)  CG: 90.8 (12.9) | EG: 89.0 (13.1)  CG: 94.3 (12.1) | EG: 87.7 (14.8)Ω  CG: 90.6 (12.7)Ω | NA | Yes (favouring the control group) |
|  | Lin *et al. 2021 [31]* | Brief Visuospatial Memory Test (BVMT)-R | Not reported | Not reported | Not reported | Not reported | No |
|  | Baik, *et al.2023 [33]* | Verbal Learning Test (VLT) | EG: 15.32 (4.46)  CG: 15.04 (4.96) | EG: 16,88 (4.00)  CG: 15.20 (5.27) | NA | NA | Yes (favouring the RCCT group) |
|  |  | Digit Span Tests (DST) | EG: 10.48 (2.58)  CG: 10.12 (2.65) | EG: 13.44 (2.68)  CG: 10.88 (3.63) | NA | NA | Yes (favouring the RCCT group) |
|  | Brill, *et al.2024 [34]* | Auditory Verbal Learning Test (AVLT) + Digit span test | Not reported | Not reported | NA | NA | No |
|  | Han, et al. 2024 [35] | Prospective and Retrospective Memory Questionnaire (PRMQ) | EG: 39.35 (7.53)  CG: 39.02 (6.96) | Not reported | Not reported |  | Yes (favouring the RCCT group) |

* Mean change for within-group differences (95% confidence interval)
** Mean (SE)
◊ Values in least square means
Ω 1 year
ϐ 16 weeks
Δ 7 months
φ 3 months

Table 2 – Methodological quality of included studies (1. Eligibility criteria; 2. Random allocation; 3. Concealment; 4. Groups similar at baseline; 5. Blinding of subjects; 6. Blinding of who administered the intervention; 7. Blinding of assessors; 8. Measures of outcome for 85% of subjects; 9. Treatment or control as allocated; 10. Statistics for between-group comparisons; 11. Point and variability measures.

| Authors (year) | 1 | 2 | 3 | 4 | 5 | 6 | 7 | 8 | 9 | 10 | 11 | Total  (2-11)* |
| --- | --- | --- | --- | --- | --- | --- | --- | --- | --- | --- | --- | --- |
| Barnes, et al 2008 [20] | 1 | 1 | 1 | 1 | 1 | 0 | 1 | 1 | 1 | 1 | 1 | 9 |
| Rosen, *et al. 2011* [21] | 1 | 1 | 1 | 1 | 1 | 1 | 1 | 1 | 1 | 1 | 1 | 10 |
| Finn, *et al 2017 [22]* | 1 | 1 | 1 | 1 | 0 | 0 | 1 | 0 | 1 | 1 | 1 | 7 |
| Hyer, *et al 2016 [23]* | 1 | 1 | 0 | 1 | 1 | 0 | 1 | 1 | 0 | 1 | 1 | 7 |
| Lin *et al. 2016 [24]* | 1 | 1 | 0 | 1 | 0 | 0 | 1 | 1 | 1 | 1 | 1 | 7 |
| Wu *et al. 2017 [25]* | 1 | 1 | 0 | 1 | 0 | 0 | 0 | 0 | 0 | 1 | 1 | 4 |
| Han, et al. 2017 [26] | 1 | 1 | 1 | 1 | 0 | 0 | 1 | 0 | 1 | 1 | 1 | 7 |
| Nousia, *et al. 2021 [27]* | 1 | 1 | 0 | 1 | 0 | 0 | 1 | 1 | 1 | 1 | 1 | 7 |
| Li *et al. 2019 [28]* | 1 | 1 | 0 | 0 | 0 | 0 | 1 | 0 | 0 | 1 | 1 | 4 |
| Manenti *et al. 2020 [29]* | 1 | 1 | 1 | 1 | 0 | 0 | 1 | 1 | 1 | 1 | 1 | 8 |
| Duff, *et al.2021 [30]* | 1 | 1 | 1 | 1 | 1 | 0 | 1 | 0 | 0 | 1 | 1 | 7 |
| Lin *et al. 2021 [31]* | 1 | 1 | 1 | 1 | 1 | 0 | 1 | 1 | 1 | 1 | 0 | 8 |
| Lin, *et al.2020 [32]* | 1 | 1 | 0 | 1 | 0 | 0 | 1 | 1 | 1 | 1 | 1 | 7 |
| Baik, *et al.2023 [33]* | 1 | 1 | 0 | 1 | 0 | 0 | 1 | 1 | 1 | 1 | 1 | 7 |
| Brill, *et al.2024 [34]* | 1 | 1 | 1 | 1 | 1 | 1 | 1 | 1 | 1 | 1 | 1 | 10 |
| Han, *et al.* 2024 [35] | 1 | 1 | 1 | 1 | 0 | 0 | 1 | 1 | 1 | 1 | 1 | 8 |
| Devanand, et al. 2022 [36] | 1 | 1 | 0 | 1 | 0 | 0 | 1 | 1 | 1 | 1 | 1 | 7 |

Legend: 0 – No; 1 – Yes: * first item of the scale not included in the total score.

Table 3 – Full search strategy for PubMed

| **Database:** PubMed |
| --- |
| **Date of search:** 7 January 2025 |
| **Search string:**  (("Mild Cognitive Impairment"[MeSH Terms] OR "Mild Cognitive Impairment"[Title/Abstract] OR MCI[Title/Abstract])  AND ("Cognitive Training"[MeSH Terms] OR "Cognitive Rehabilitation"[MeSH Terms]  OR "Cognitive training"[Title/Abstract] OR "Cognitive rehabilitation"[Title/Abstract]  OR "Computerized cognitive training"[Title/Abstract] OR "Digital cognitive training"[Title/Abstract]  OR "Virtual reality"[Title/Abstract] OR "Serious games"[Title/Abstract]  OR "Web-based"[Title/Abstract] OR "Mobile"[Title/Abstract])  AND ("Randomized Controlled Trial"[Publication Type] OR "randomized controlled trial"[Title/Abstract] OR RCT[Title/Abstract])) |
| **Filters applied:**   - Humans - Age: 60 years and older |
| Article type: Exclude reviews, systematic reviews, and books   - Language: English |
